# Prevalence of Ocular Injuries Following Road Traffic Accidents Among Two-Wheeler Riders/Pillions and the Factors Influencing Them Reported in a Tertiary Care Center in Chengalpattu in Tamil Nadu, India

**DOI:** 10.7759/cureus.72470

**Published:** 2024-10-27

**Authors:** Maduraa Devandan, Lily Daniel, Anila Britta Sanjay

**Affiliations:** 1 Ophthalmology, SRM Medical College and Hospital, Chennai, IND; 2 Oral and Maxillofacial Surgery, SRM Medical College and Hospital, Chennai, IND

**Keywords:** loss of vision, ocular injury, road traffic accident, stringent regulation, visual acuity

## Abstract

Introduction

Road traffic accident-related ocular damage is a serious public health issue that can result in unilateral blindness. Several individual risk factors lead to ocular injury and various levels of visual impairment.

Objectives

The objective is to assess the demography and pattern of ocular, orbital, and eyelid injuries among motorcycle accident cases attending a medical college and hospital in Chengalpattu district Tamil Nadu India.

Methodology

A cross-sectional study was conducted among 67 victims of road traffic accidents who had sustained ocular injuries from October 2023 to June 2024. The selected participants were subjected to a questionnaire regarding the details of the history of events leading to the road traffic accident, and complete ophthalmic examination and radiological investigations were carried out. Informed consent was obtained from all the participants, and in cases where the participant was unable to give consent, informed consent was obtained from their family members. The collected data were analyzed using SPSS version 21 (IBM Corp., Armonk, NY).

Result

The majority (76.1%) were male between the age group of 31-40 years, and they were all motor vehicle/cycle riders seated in the front. Most accidents happened in rural (68.7%), highways (52.2%), and the driver being under the influence of alcohol (64.2%). Most of the participants (80.6%) did not use helmets, and 19.4% were using mobile phones while driving. Visual acuity was affected in 57% and 41.8% suffered from orbital floor fracture. About 92.5% of the participants sustained subconjunctival hemorrhage with ecchymosis and 31.34% had lacerations of the upper eyelid with periorbital edema. Among the participants, 9% had a loss of vision due to vitreous hemorrhage and 9% due to Berlin’s edema.

Conclusion

Middle-aged males driving two-wheelers on rural highways, those who did not wear helmets, and those who were under the influence of alcohol were the vulnerable group. It is advised that both front and back-seated persons use safety precautions, such as helmets, and avoid placing the child in front of the rider while driving. Stringent regulations designed to improve conformity to traffic rules are essential in preventing ocular injuries.

## Introduction

Road traffic accidents have emerged as a major public health problem in the recent past. It has led to an increased rate of mortality and morbidity following the recent development in the lifestyle and automobile industry [1]. Road traffic accidents are one of the top 10 causes of death globally. According to a recent publication from WHO, India contributes to 11% of mortality due to accidents globally and the rate will increase over the years due to an increase in the growing number of vehicles [2].

In addition to the increasing number of vehicles, factors, such as knowledge and compliance with traffic rules, poor traffic management and quality of the road, an increasing number of new untrained drivers, and high-risk behavior like drunken driving, stunts, racing, etc., increase the morbidity and mortality [3]. The avoidance of brain injuries, spine injuries, thoracic injuries, abdominal injuries, long bone fractures, pelvic injuries, and ear, nose, and throat injuries are a major area of attention for reducing consequences, from road traffic accidents [4]. Often prevention measures focusing on ocular injuries are neglected. A grievous injury to the eye might cause sight impairment and make it difficult for the victim to go about their regular life. Ocular trauma, on its whole, is the major cause of uniocular complete and partial loss of vision, accounting for over 500,000 cases of blindness worldwide [5]. According to the data on head injuries following trauma, 25% to 30% of head injuries lead to visual and ocular defects. Ocular injuries in road traffic accidents are the leading cause of all traumatic ocular injuries [6].

India boasts one of the world's biggest road networks. As a result, our nation's transportation system has undergone numerous modifications [7]. There are far more motorcycles on the road now than there used to be, primarily due to government-sponsored programs, increasing population, better financial conditions for the residents of rural areas, and easier access to bank vehicle loans [8].

In India, as in most of Asia and other emerging nations, motorcycles are the most widely used form of transportation in both rural and urban regions [9]. Accident risk is increased in rural locations by the absence of bike tracks, service lanes, and footpaths where non-motorized transportation coexists with motorized traffic [10]. Hence, given its detrimental effects on the economy, public health, and welfare of the populace, road safety is a national priority [11].

Ocular injury findings in road traffic accidents include laceration of eyelids, abrasions of the cornea and conjunctiva, scleral tear, perforation of the globe with lacrimal gland injury, fracture of the orbital wall, and ocular muscle injury [12]. Road traffic incidents that cause traumatizing ocular injuries may cause some form of sight impairment, which will put a significant financial strain on both the affected person and the community [13]. Patterns and severity of eyelid and ocular injuries due to environmental-related factors are studied here which adds to the novelty. The results of this study will help understand the distribution and severity of ocular injuries among motorcycle accident victims, and they will provide the cornerstone for the development and execution of preventive actions by relevant authorities.

## Materials and methods

This cross-sectional study was conducted at the casualty/emergency department in SRM Hospital, Chengalpattu, South India, for a period of eight months from October 2023 to June 2024. This study involved people of both sexes from rural and urban areas with a history of ocular injuries following road traffic accidents. The sample size was calculated based on the prevalence of ocular trauma findings, following road traffic accidents which was 57.93% with a relative precision of 25% and 95% confidence interval level based on the study by Shtewi et al. (Libya) [14]. The final estimated sample size including a 10% non-response rate was 67. The selection of participants strictly followed the inclusion and exclusion criteria until the desired sample size was achieved.

The study aims to assess the demography and pattern of ocular, orbital, and eyelid injuries among motorcycle accident cases attending a medical college, hospital in Chengalpattu district, South India. The objectives were to assess the agent-host environmental factors associated with motorcycle accident cases and the correlation of the pattern of ocular injuries associated with the nature of the accident reported in a medical college hospital in the Chengalpattu district, Tamil Nadu, South India.

Inclusion criteria of the participants were patients attending casualty/emergency department following two-wheeler motorcycle accidents, in the age group of five to 45 years including both sexes and patients with no underlying corneal and retinal pathologies. Exclusion criteria were age more than 45 years, patients with pre-existent corneal pathology, optic nerve head pathology, retinal disorders, congenital color blindness, and cataracts (based on past medical history and clinical findings). Eighteen cases were excluded based on age criteria as they were above 45 years and had pre-existing immature cataracts and one case was excluded due to known glaucomatous optic atrophy.

The selected participants were given a questionnaire or asked questions related to their socio-demographic profile, history of road traffic accidents, and mode of injury. The blood pressure, pulse rate, and heart rate were checked in the casualty at the time of admission and the findings were noted. Following this general examination and detailed ophthalmic examination were done, with available study tools in the casualty. The type of injury and extent of injury were assessed, which was correlated with the environmental factors leading to injury and safety measures/precautions taken while riding or violation of traffic rules. Visual acuity was checked (bedside counting of fingers)/Snellen chart and anterior segment findings were noted. Pupillary response and extraocular movements were carefully monitored. Fundus examination was done after dilating the pupils with tropicamide eye drops. Radiological investigations were done using computed tomography of orbit/computed tomography of facial bones and magnetic resonance imaging, wherever needed, to find out the severity of the injury.

The data of the study participants were entered in Microsoft Excel (Microsoft Corporation, Washington, DC) and verified for errors and data cleaning was done by peers. The data was analyzed using SPSS version 21 (IBM Corp., Armonk, NY). Data analysis was performed using descriptive statistics to summarize the demographics, injury types, and visual acuity. The chi-square test was used to compare the severity of injuries with age and sex distribution and p value of <0.05 was considered to be statistically significant. Frequency distributions and percentages were calculated for categorical variables.

Informed consent was obtained from all patients and consent for children less than 18 years old was obtained from their parents/guardians. Official permission to conduct this study was obtained from the Institutional Ethics Committee of the local institution (SRM Medical College and Hospital, Chennai, SRMIEC-ST0723-582).

## Results

The right eye was involved in (62.7%), while the left eye was involved in 25 cases (37%) (Table 1).

**Table 1:** Distribution of demographics and motor vehicle accident characteristics of study participants

Characteristics	Frequency (N=67)	Percentage (%)
Age		
5-10	5	7.5
11-20	4	6.0
21-30	14	20.9
31-40	28	41.8
41-45	16	23.9
Gender		
Female	16	23.9
Male	51	76.1
Type of road		
Bridge	12	17.9
Highway	35	52.2
Mud road	20	29.9
Area		
Rural	46	68.7
Urban	21	31.3
Under the influence of cannabis		
No	65	97
Yes	2	2.98
Under the influence of alcohol		
No	24	35.8
Yes	43	64.2
Riding on the wrong side of the road		
No	45	67.2
Yes	22	32.8
Driver or passenger		
Driver	47	70.1
Passenger	20	29.9
Seated position on two-wheeler		
Front	47	70.1
Back	20	29.9
Helmet use		
No	54	80.6
Yes	13	19.4
Mobile phone use		
No	54	80.6
Yes	13	19.4
Listening to music while driving		
No	47	70.1
Yes	20	29.9
Driver’s license		
No	10	14.9
Yes	57	85.1
Collision with another vehicle/road divider		
No	48	71.6
Yes	19	28.4
Accident due to animal		
No	58	86.6
Yes	9	13.4
Object impact on eyes/head		
No	48	71.6
Yes	19	28.4
Object penetration in eyes		
No	63	94.0
Yes	4	6.0
Presence of children in front		
No	61	91.0
Yes	6	9.0
Overtaking vehicle		
No	56	83.6
Yes	11	16.4
Blood pressure		
Elevated	11	16.4
Hypotension	6	9.0
Normal	50	74.6

Severe sight-threatening injuries were less common with only perception of light and projection of rays present among 6% of the affected individuals, and 3% of cases had complete loss of vision. The majority of the subject's visual acuity changes were negligible or normal. Severe sight-threatening injuries were less common (6%), and 3% of cases had complete loss of vision (Table 2). Color vision was affected in 20.9% of the patients, which is shown in Table 2.

**Table 2:** Distribution of study participants according to visual acuity and color vision of affected eye

Unaided visual acuity and color vision of affected eye	Frequency (N=67)	Percentage (%)
6/6	21	31.3
6/9	8	11.9
6/12	10	14.9
6/18	3	4.5
6/24	5	7.5
6/36	5	7.5
6/60	4	6.0
< 6/60	2	3.0
No perception of light (NPL)	2	3.0
Perception of light + (PL +), projection of rays absent in lateral, superior and inferior directions, projection of rays present in medial direction	3	4.5
Only PL+ PR+	4	6.0
Color vision of affected side		
Normal	53	79.1
Impaired	14	20.9

**Figure 1:**
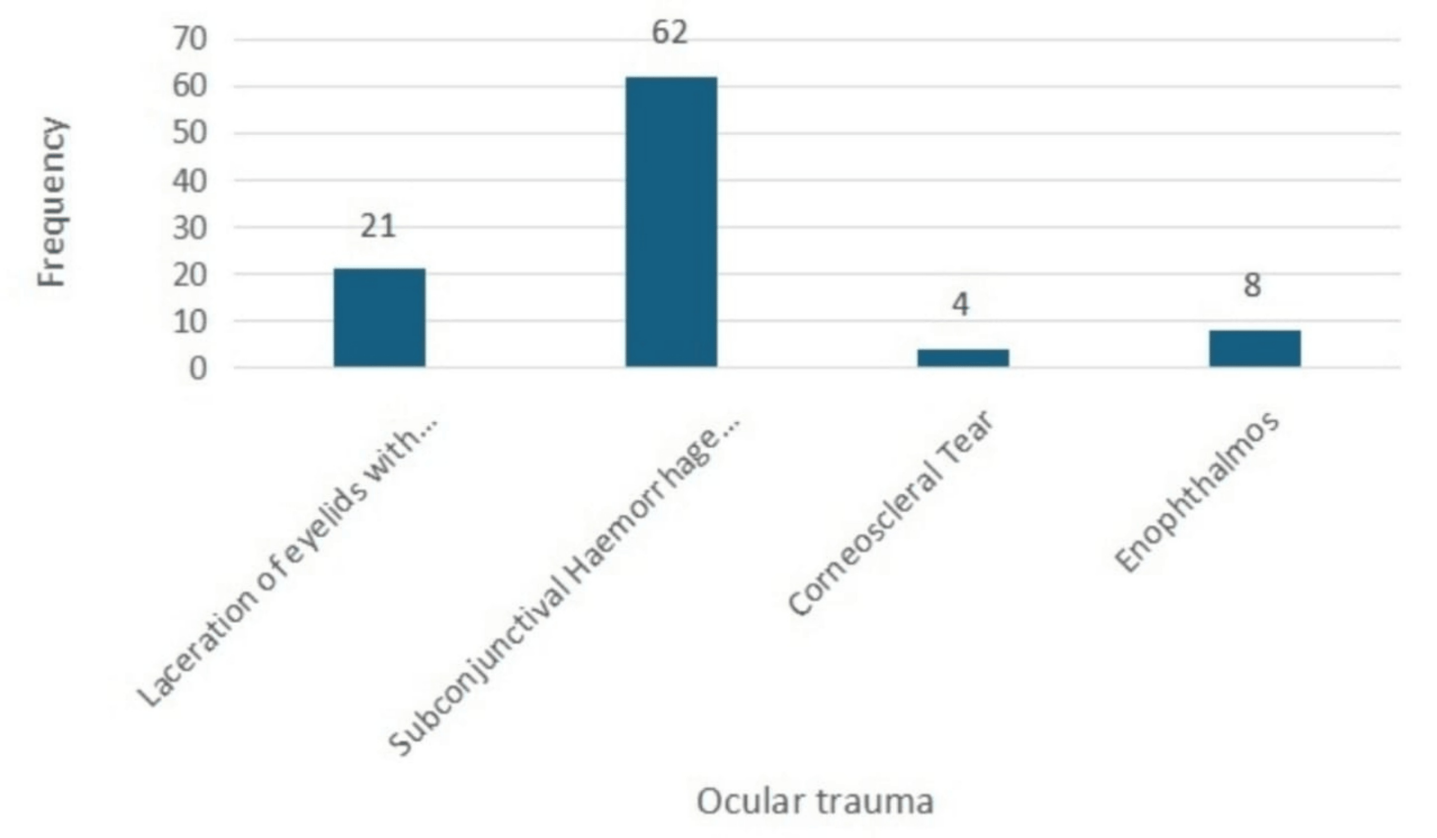
Frequency of ocular trauma findings in the patients

The anterior segment injuries are shown in Table 3 and depicted in Figure 1. About 62 (92.5%) presented with subconjunctival hemorrhage. The intraocular pressure (IOP) was elevated in about 14 patients (20.9%). Approximately 21 patients (31.3%) had upper eyelid lacerations with periorbital edema, 41.8% had orbital floor fracture and, 32% had a lateral wall with orbital floor fracture. Enophthalmos was noted in eight patients (11.9%) with associated orbital floor fracture. 

**Table 3:** Anterior segment findings as a result of road traffic accident

Ocular Injury findings	Frequency (N=67)	Percentage (%)
Extraocular muscle (EOM) restriction	7	10.4
Intraocular pressure (IOP) elevation	14	20.9
Laceration of eyelids with periorbital edema	21	31.34
Subconjunctival hemorrhage (SCH) with ecchymosis	62	92.5
Corneal tear	4	6.0
Corneoscleral tear	4	6.0
Hyphemia	3	4.5
Lens dislocation/subluxation	2	3.0
Globe perforation	1	1.5
Foreign body penetration	2	3.0
Enophthalmos	8	11.9
Sphincter tear	1	1.5
Relative afferent pupillary defect (RAPD)	7	10.4

On the other hand, posterior segment findings were noted in 9% of individuals who had vitreous hemorrhage, 9% had Berlin's edema, and one patient (1.5%) had retinal detachment, as shown in Table 4. Traumatic optic neuropathy with relative afferent pupillary defect of the affected eye was noted in four patients (6%) which is shown in Table 4. Imaging results showed that six participants (9%) had acoustic hyperdensity in the vitreous cavity, which indicated the presence of vitreous hemorrhage, and one patient (1.5%) had a lens dislocated into the vitreous cavity, which was the B scan findings. 

**Table 4:** Causes of vision loss due to posterior segment pathology as a result of trauma

Posterior segment ocular injury findings	Frequency (N=67)	Percentage (%)
Berlin's edema	6	9.0
Retinal detachment	1	1.5
Vitreous hemorrhage	6	9.0
Traumatic optic neuropathy	4	6.0
Choroidal tear	1	1.5

Table 5 compares the driving behavior with using mobile phones, without a helmet, and under the influence of alcohol with age category distribution. The chi-square test was used. Statistically significant at a 5% level of significance. The p-value was statistically significant (p<0.001) for people who had consumed alcohol leading to an accident. Among those who were driving under the influence of alcohol and sustained sight-threatening injuries, were in the age groups of 21-30 years and 41-45 years. On the other hand, the p-value was not statistically significant for variables, listening to music (p=0.22), using mobile phones (p=0.28), and not wearing a helmet (p=0.831).

**Table 5:** Driving behavior and safety equipment usage by age category

Variables		Age category					Total	P-value
		5-10	11-20	21-30	31-40	41-45		
Were you wearing a helmet	No	3	4	11	22	14	54	0.831
	Yes	1	0	3	5	2	11	
Were you using mobile phone	No	4	2	13	21	12	52	0.284
	Yes	0	2	1	6	4	13	
Were you listening to music while driving?	No	4	4	11	16	10	45	0.22
	Yes	0	0	3	11	6	20	
Were you under the influence of alcohol while driving	No	5	3	0	14	2	24	<0.001
	Yes	0	1	14	14	14	43	

Table 6 shows the severity of ocular injury findings with age category distribution. The chi-square test was used. Statistical significance was taken to be p<0.05. Here all the anterior segment ocular injuries were equally present among all age groups in our study. Subconjunctival hemorrhage was most common among the 31-40 years age group, but the p-value (p=0.813) was not statistically significant. Posterior segment findings which included berlins edema, vitreous hemorrhage, and retinal detachment contributed to significant vision loss, the severity being more and the p-value was statistically significant (p<0.021).

**Table 6:** Ocular injury findings by age category

Ocular Injury findings		Age category					Total	P-value
		5-10	11-20	21-30	31-40	41-45		
Sub conjunctival hemorrhage	No	0	0	2	2	1	5	0.813
	Yes	4	4	12	25	15	60	
Corneal tear	No	4	4	13	25	15	61	0.961
	Yes	0	0	1	2	1	4	
Corneo-scleral tear	No	4	4	12	26	15	61	0.661
	Yes	0	0	2	1	1	4	
Enophthalmos	No	4	3	12	24	14	57	0.871
	Yes	0	1	2	3	2	8	
Any foreign body penetration	No	4	4	13	26	16	63	0.933
	Yes	0	0	1	1	0	2	
Traumatic optic neuropathy	No	4	4	13	24	15	60	0.884
	Yes	0	0	1	3	1	5	
Choroidal tear	no	4	4	13	27	16	64	0.448
	Yes	0	0	1	1	0	1	
Sphincter tear	no	4	4	14	26	16	64	0.839
	Yes	0	0	0	1	0	1	
Posterior segment findings	Number of patients without posterior segment findings	3	2	10	23	14	52	0.021*
	Berlins edema+	1	1	1	1	2	6	
	Retinal detachment+	0	1	0	0	0	1	
	Vitreous hemorrhage	0	0	3	3	0	6	

Table 7 shows the severity of ocular injury findings among males and females, and the Chi-squared test was used to compare the variables. An equal proportion of ocular injury findings was noted among both sexes and the p-value was not statistically significant for both anterior and posterior segment injury findings. Posterior segment injuries (p=0.854) were not statistically significant.

**Table 7:** Ocular injury findings by sex

Ocular Injury findings		Sex		Total	P-value
		Female	Male		
Sub conjunctival hemorrhage	No	0	5	5	0.193
	Yes	16	46	62	
Corneal tear	No	14	49	63	0.206
	Yes	2	2	4	
Corneo -scleral tear	No	16	47	63	0.248
	Yes	0	4	4	
Enophthalmos	No	14	45	59	0.937
	Yes	2	6	8	
Foreign body penetration	No	15	50	65	0.585
	Yes	1	1	2	
Traumatic optic neuropathy	No	14	48	62	0.379
	Yes	2	3	5	
Iris sphincter tear	no	15	51	66	0.072
	Yes	1	0	1	
Choroidal tear	No	16	50	66	0.573
	Yes	0	1	1	
Posterior segment injuries	Number of patients with no posterior segment injuries	13	41	54	0.854
	Berlins edema+	1	5	6	
	retinal detachment+	0	1	1	
	Vitreous hemorrhage	2	4	6	

Table 8 compares the driving behavior related to listening to music, mobile usage, and lack of helmet use among males and females using the Chi-square test. Statistical significance was considered to be <0.05. Driving under the influence of alcohol was statistically significant among males and females (p<0.001) where more males than females as most of the males were under the influence of alcohol when the accident occurred in our study. On the other hand, more females violated the rules by driving on the wrong side of the road and sustained ocular injuries, but the p-value was not statistically significant (p=0.287). In our study, more males were listening to music leading to accidents and ocular trauma, but the p-value was not statistically significant (p=0.443).

**Table 8:** Driving behavior and safety equipment usage by sex

Variables		Sex		Total	P-value
		Female	Male		
Were you wearing a helmet	No	13	41	54	0.94
	Yes	3	10	13	
Were you using mobile phone	No	11	43	54	0.17
	Yes	5	8	13	
Were you listening to music while driving?	No	10	37	47	0.443
	Yes	6	14	20	
were you driving on the wrong side of road	No	9	36	45	0.287
	Yes	7	15	22	
Were you under the influence of alcohol while driving	No	14	10	24	<0.001
	Yes	2	41	43	

## Discussion

Ocular injuries following road traffic accidents are often a neglected condition in the health care system. Many incidents have gone unnoticed leading to late complications [12]. We have assessed the profile of the ocular injuries reported in the casualty following road traffic accidents in SRM hospital in Tamil Nadu, India.

In our study, the prevalence of ocular injuries is in the age group 30 to 40 years, male sex, rural habitat, highway, and use of alcohol prior to driving were vulnerable to injuries. Most of those affected were not wearing helmets and were listening to music. Similar to our findings, the study by Mayura et al. in Uttar Pradesh stated that most patients were in the age group 30 years, male sex were from rural habitats, most accidents happened on highways, lacked protective gear, and had other distractions while driving [15]. The study by Nalukenge et al. in Uganda had different findings from our study, where only age, sex, place of accident, and locality were similar to our findings [16]. However, the majority of accidents in their study occurred in those not under the influence of alcohol and those with protective gear. The global prevalence of ocular injury following road traffic accidents is identical. The influence of alcohol depends on access to it and compliance with traffic rules.

The events leading to ocular injury among the study participants had varied results. Most of them did not have direct impact or penetrating injury to the eye while they were driving in the same lane and for most of them the affected side was the right eye. Similar findings were observed in the studies conducted by Akhiwu et al. in Nigeria, and Wagh et al. in Central India [17,18]. In contrast to our findings, in the study by Sahu et al. in India the prevalence was higher in the left eye, but the type of injury was similar. Their study stated that protective gear will reduce injury to the eye which correlated with our study [19]. In 15% of the patients, the cause of injury was due to a direct collision with another vehicle due to traveling on the wrong side of the road which resulted in an orbital floor fracture with periorbital edema. In 9% of the patients, the cause for periorbital edema with laceration of the upper eyelid was attributed to the use of a mobile phone while driving leading to a collision with another vehicle.

Following trauma, one-third of them had normal visual acuity while the majority of participants experienced diminished vision, and the color vision was impaired in less than a quarter of them. Out of 20.9% of the patients with impaired color vision, the cause in 6% of the patients was traumatic optic neuropathy, while the cause for reduced visual acuity and impaired color vision among the others was due to vitreous hemorrhage (6%), berlins edema (6%), globe perforation with choroidal tear (1.5%), and retinal detachment. Orbital floor and lateral wall orbital bone fractures were noted in 32% of the participants who sustained injuries. One of the patient's open globe injuries was caused by a cattle accident that resulted in a fall from the vehicle, which allowed a sharp object to pierce the eye leading to grievous injury. Blood was noted in the anterior chamber, and on the B scan, the lens was dislocated into the vitreous cavity. The patient was driving on the wrong side of the road with no safety precautions such as a helmet which led to severe impact to the visual recovery. Similar to our study findings, observations from the studies by Sahu et al. in India and Padmaraj et al. in India were identical. These findings also indicate the lack of personal protective measures like helmets was the major factor for trauma to the orbit leading to severe impact on the vision [19,20].

In our study, two participants used cannabis while riding the vehicle. One among them had a severe injury from a direct hit on the road divider and foreign body penetration into the eye, resulting in a globe perforation and a corneoscleral tear. The damage was significant enough to involve the posterior segment of the eye, leading to choroidal detachment which was diagnosed in the B-scan that severely affected his visual recovery. Our study is compared with the study by Mark Asbridge where he concluded that there is a correlation between cannabis use and a higher chance of vehicle collision, particularly fatal ones from the results obtained from his study “Acute cannabis consumption and motor vehicle collision risk-systematic review of observational studies and meta-analysis" [21].

Following any trauma to the orbit or eye the most common injury to the anterior segment included sub-conjunctival hemorrhage and ecchymosis (92.5%) followed by laceration of eyelids with periorbital edema. Raised intraocular pressure was noted in 7% of patients with orbital floor fracture, in 3% of patients the cause for elevated intraocular pressure was due to blood in the anterior chamber, and in one patient raised intraocular pressure was associated with orbital floor fracture and traumatic optic neuropathy. Studies by Shtewi et al. in Libia, Krishna et al. in India, and Nalukenga et al. in Uganda had similar findings of high prevalence of subconjunctival hemorrhage during road traffic accidents. Similar to our findings, these studies had ecchymosis as the second most common manifestation following trauma. All these articles hypothesize that lack of protective equipment while riding or traveling in two-wheelers is the major cause of ocular injuries [14,16,22].

Loss of vision is a serious morbidity following a trauma to the eye. The most common cause of ocular morbidity and loss of vision was road traffic accidents. Among the study participants, five were children less than 10 years old who were seated in front of the driver. They sustained severe ecchymosis to the affected eye with floor fracture of the orbit and zygomatic bone fracture which needed immediate surgical intervention with the oral maxillary facial surgery team. Furthermore, among our study participants, one-fifth presented with a sudden loss of vision due to vitreous hemorrhage and Berlin's edema as a direct result of running into the road divider. Different studies identified different causes of blindness following trauma. One of the equivocal findings was vitreous hemorrhage while the other causes were perforation, retinal detachment, and lens dislocation in the studies by Frederick et al., Bokhari et al. in India, and Asbridge et al. in systemic review and meta-analysis. All findings indicate that vitreous hemorrhage is the most common cause of blindness following trauma and protective gear is a must while riding a two-wheeler [21,23,24]. The cause for traumatic optic neuropathy among three patients in our study was determined to be a fall from a two-wheeler after it collided with another vehicle, which resulted in injury to the occipital region of the head because the patients were not wearing helmets. Appropriate treatment was administered to these patients who presented with a relative afferent pupillary defect on the affected side associated with traumatic optic neuropathy.

The majority of the potentially fatal injuries in our study were brought on by trauma sustained while driving while intoxicated, which was prevalent among those aged 20 to 45 and the majority were males. The primary causes of this conduct were the low cost of liquor, which is frequently found in rural regions, and alcohol addiction among college students, which resulted in them breaking the rules when driving. The majority of those in the 30-40 age range who broke traffic laws by using cell phones while driving were in the same age range as the 80.6% of participants in our study who did not wear helmets when driving. It was discovered that this behavior was the result of these people's ignorance to abide by rules and lack of awareness to use protective equipment while driving.

Recommendation

Public awareness regarding collisions with another vehicle due to traveling on the wrong side of the road the importance of wearing helmets and the use of protective goggles made of polycarbonate material for youngsters and middle-aged people should be implemented. Strict protocol on ophthalmic assessment following all road traffic accidents should be implemented at the casualty and emergency rooms in the healthcare facilities. Special care should be provided to those road traffic accident victims with head and face injuries. The traffic rules and regulations towards speed monitoring, use of protective gear, and policies against riding under the influence of alcohol should be made stringent and violators should be penalized heavily. Quality roads with separate lanes for two-wheeler riders should be made mandatory and restriction of two-wheelers in highways should be advocated. The speed limit for two-wheelers on highways and in rural areas should be brought down. Consistent with the study, it has been observed that placing a child in front of the rider on a two-wheeler can lead to zygomatic and orbital floor fractures with sight-threatening injury. It is crucial to establish and enforce regulations that ensure a child's safe positioning on a two-wheeler, with the use of proper protective headgear.

Study limitations

Our study is a cross-sectional study conducted in a single health care facility which leads to a lack of generalization. It involved patients who reported in the casualty/emergency department with a history of ocular trauma from road traffic accidents in two-wheelers. Our study excluded road traffic accidents from other modes of transportation. Our study could have missed those patients with major injuries who were shifted to other wards based on their needs. In our study the immediate visual acuity/color vision assessment and extraocular movement findings could not be recorded in patients who were unconscious after the accident and for these patients the findings were assessed later during recovery. In our study, blood pressure readings were taken as soon as the patient was admitted to the emergency room. As a result, it was not possible to conclude whether the raised blood pressure was due to the accident or due to pre-existing hypertension. This study did not include long-term follow-up of patients, so the best corrected visual acuity after recovery could not be assessed. Moreover, it was only a short time study of nine months, so this time frame could have had a difference in the prevalence of ocular injuries.

## Conclusions

Ophthalmic injuries were more common in middle-aged males, who sustained injuries to the face and head in road traffic accidents in rural and on highways. Risk factors for ocular injuries include being under the influence of alcohol, rider of a two-wheeler, using a mobile phone while riding, and lack of protective gear like a helmet while riding the two-wheeler. The majority of injuries sustained were non-penetrating. One-third of them had normal visual acuity while most of them had visual acuity of various degrees.

The majority of the patients in our study sustained orbital floor fractures with raised intraocular pressure, and one-third of the patient's orbital floor fractures were associated with enophthalmos. In addition to it, most of them sustained subconjunctival hemorrhage and ecchymosis. Some of them had loss of vision following road traffic accidents and the most common cause for visual loss in our study was due to vitreous hemorrhage, whereas in 6% the cause for loss of vision was traumatic optic neuropathy. The majority of the ocular injuries in our study were sustained due to an accident caused by a collision with another vehicle, while driving at a higher speed in rural areas than the recommended speed limit, without the use of a helmet, and under the influence of alcohol.

## Appendices

Dr. Maduraa Devandan,                      Dr. Lily Daniel

 PROFORMA  

  Name:

  UHID NO                           Age                             sex                     Contact Details

 Type of RTA

 Type of road- Highway/Bridge/Mud Road

 Questionnaire

1.   Were you the driver or the pillion? Yes /No?

2.   were you under the influence of alcohol? Yes /No

3.  Were you driving on the wrong side of the road? Yes /No 

4.   Were you on drugs when the accident occurred? Yes/No (If so mention the drug)

5.  At what speed did you drive? 

6.  Were you wearing a helmet? Yes/No.

7.  Were you using a mobile phone (talking) while driving?  Yes/No.

8.  Were you listening to music while driving? Yes/No

9.  Did the driver have a license? Yes/ No

10.  Did the rider fall over a stray dog or cattle/ or was the accident due to bump over an animal which came in your way? Yes/No 

11.  Did you directly bump into a road divider or another vehicle? Yes/No 

12. Did any other object fall over your eyes /head? Yes /No 

13.   Did any object penetrate your eyes? Yes/no 

14. Were there any children in front of you while riding and was it the reason for the accident? Yes/No

15.  Were you trying to overtake any vehicle? Yes /No

This is an original research article, and the questionnaire was prepared by the authors Dr. Maduraa and Dr. Lily Daniel after observing two-wheeler accident cases in SRM Hospital and the environmental factors leading to two-wheeler accidents.

## References

[1] Sharma S, Patnaik L, Mohanty S, Sahu T (2024). An epidemiological study on road traffic accidents at a tertiary care hospital of eastern India [Internet]. https://www.semanticscholar.org/paper/An-Epidemiological-Study-on-Road-Traffic-Accidents-Sharma-Patnaik/729603c003b7afb2916e8197003f8fe2c6ee2fe2.

[2] (2024). Road traffic injuries. https://www.who.int/news-room/fact-sheets/detail/road-traffic-injuries.

[3] Raina S, Awasthi B, Verma L (2019). Epidemiological determinants of road traffic accidents in a largely rural hilly population. J Sci Soc [Internet.

[4] Hareru HE, Negassa B, Kassa Abebe R (2022). The epidemiology of road traffic accidents and associated factors among drivers in Dilla Town, Southern Ethiopia. Front Public Health.

[5] K S, Karanth SS (2019). Clinical profile of ocular injuries following road traffic accidents in a tertiary care centre. Indian J Clin Exp Ophthalmol.

[6] Pizzarello LD (1998). Ocular trauma: time for action. Ophthalmic Epidemiol.

[7] Kuhn F, Collins P, Morris R, Witherspoon CD (1994). Epidemiology of motor vehicle crash-related serious eye injuries. Accid Anal Prev.

[8] Maurya RP, Srivastav T, Singh VP, Mishra CP, Al-Mujaini A (2019). The epidemiology of ocular trauma in Northern India: a teaching hospital study. Oman J Ophthalmol.

[9] Georgouli T, Pountos I, Chang BY, Giannoudis PV (2011). Prevalence of ocular and orbital injuries in polytrauma patients. Eur J Trauma Emerg Surg.

[10] Kumar S, Mahima Mahima, Srivastava DK, Kharya P, Sachan N, Kiran K (2020). Analysis of risk factors contributing to road traffic accidents in a tertiary care hospital. A hospital based cross-sectional study. Chin J Traumatol.

[11] Saeednejad M, Sadeghian F, Fayaz M (2020). Association of social determinants of health and road traffic deaths: a systematic review. Bull Emerg Trauma.

[12] (2024). Ocular trauma prevention strategies and patient counseling. https://pubmed.ncbi.nlm.nih.gov/35593844/.

[13] Willmann D, Fu L, Melanson SW (2023). Corneal Injury.

[14] Shtewi ME, Shishko MN, Purohit GK (1999). Road traffic accidents and ocular trauma: experience at Tripoli eye hospital, Libya. Commun Eye Health.

[15] Maurya RP, Singh VP, Mishra CP (2021). Eye injuries in motor vehicle accidents: epidemiology, spectrum of injury and analysis of risk factors. Int J Ocul Oncol Oculoplasty.

[16] Nalukenge C, Sebabi FO, Okello B (2023). Factors associated with ocular injuries among adult road traffic accident patients presenting at Mulago National Referral Hospital, Uganda. Afr Health Sci.

[17] Panshak TE, Akhiwu BI, Ramyil AV, Saleh N, Wade P, Ladeinde AL, Mpyet C (2022). Ophthalmic injuries in patients with maxillofacial trauma presenting to a teaching hospital in North Central Nigeria. J West Afr Coll Surg.

[18] Wagh V, Tidake P (2022). Clinical study and profile of ocular trauma: findings from a rural hospital in central India. Cureus.

[19] Sahu SK, Radhakrishnan RV, Mohanty CR (2024). Pattern and clinical profile of patients with ocular trauma presenting to the emergency department of a teaching hospital in India: a prospective observational study. Turk J Emerg Med.

[20] Padmaraj JM, Shetgar AC, Mallkarjunaswamy DL, Ramanna D, Venkatesh RH (2020). Ocular injuries following road traffic accidents: a hospital based case series study. Indian J Clin Exp Ophthalmol.

[21] Asbridge M, Hayden JA, Cartwright JL (2012). Acute cannabis consumption and motor vehicle collision risk: systematic review of observational studies and meta-analysis. BMJ.

[22] Krishna R (2023). A study on the ocular manifestations of road traffic accident cases attending government regional eye hospital. Asian J Pharm Clin Res.

[23] Frederick M, Kumaresan V, Anuradha P (2022). A clinical study on types of ocular injuries following road traffic accidents in a tertiary health care hospital in Chennai, Tamil Nadu. Int J Adv Med.

[24] Bokhari SS, Sujatha RMA (2021). Ocular manifestations in road traffic accidents. Int J Ocul Oncol Oculoplasty.

